# The Somatosensory Cortex and Body Representation: Updating the Motor System during a Visuoproprioceptive Cue Conflict

**DOI:** 10.1523/JNEUROSCI.0181-25.2025

**Published:** 2025-07-09

**Authors:** Jasmine L. Mirdamadi, Reshma Babu, Manasi Wali, Courtney R. Seigel, Anna Hsiao, Trevor Lee-Miller, Hannah J. Block

**Affiliations:** Department of Kinesiology, School of Public Health-Bloomington, Indiana University, Bloomington, Indiana 47405

**Keywords:** body representation, cue conflict, multisensory, proprioception, recalibration, somatosensory cortex

## Abstract

The brain's representation of hand position is critical for voluntary movement. Representation is multisensory, combining visual and proprioceptive cues. When these cues conflict, the brain recalibrates its unimodal estimates, shifting them closer together to compensate. Research suggests such updates to body representation are communicated to the motor system to keep hand movements accurate. The neural mechanism is unclear and may depend on how the brain integrates and recalibrates visuoproprioceptive signals: models ranging from hierarchical convergence after unisensory processing to more distributed frameworks have been proposed. We hypothesized that the primary somatosensory cortex (S1) is crucial in this updating process due to its role in proprioception and connections with both primary motor cortex (M1) and multisensory regions of the posterior parietal cortex (PPC). In human participants of both sexes, Experiment 1 showed that short-latency afferent inhibition, a measure of somatosensory–motor integration, changed with proprioceptive recalibration and only in the presence of a cue conflict. This indicates that S1 activity reflects the results of multisensory computations and is inconsistent with a pure hierarchical convergence model of visuoproprioceptive integration. Experiment 2 found that modulating S1, but not M1, with repetitive transcranial magnetic stimulation increased proprioceptive variance and recalibration. This is consistent with the idea that motor effects of proprioceptive recalibration are mediated by the S1→M1 pathway, although not directly controlled by M1 itself. The specificity of our findings to proprioceptive—not visual—recalibration argues against a fully distributed framework, but our findings support a model of multisensory integration with reciprocal interactions between S1 and PPC.

## Significance Statement

Representation of the hand, which is critical for accurate control of movement, comes from weighting and combining available proprioceptive (position sense) and visual cues. Our results suggest that when the hand representation is modified by introducing a conflict between these cues, the motor system receives updates directly from the primary somatosensory cortex (S1). These updates are specific to the change in proprioceptive representation and are absent when cues are not in conflict, suggesting S1 activity reflects the result of visuoproprioceptive computations. This is inconsistent with a hierarchical convergence model of multisensory integration but could reflect reciprocal interactions between S1 and multisensory regions of the posterior parietal cortex.

## Introduction

Performing complex movements with the hands is a universal human experience. By adulthood, we have learned countless such motor skills: tying our shoes, controlling a computer mouse, etc. Understanding how the brain learns such skills is challenging due to the interplay of motor, sensory, and multisensory processes. “Learning” entails not only motor system refinements but also fine-tuning perception of the hand itself. When the hand is visible, its perceived location reflects a weighted average of visual and proprioceptive cues (Ghahramani et al., 1997; van Beers et al., 1999). If these cues conflict but are still interpreted as arising from the same source (the hand), the brain is expected to recalibrate each unimodal estimate in proportion to its relative variance, updating the integrated multisensory estimate accordingly (Ghahramani et al., 1997).

Visuoproprioceptive recalibration in hand perception is an example of the brain's ability to update body representation. Converging evidence from research in perception (Botvinick and Cohen, 1998; Kammers et al., 2009), behavior (Cressman and Henriques, 2010; Ruttle et al., 2018; Mostafa et al., 2019), and neurophysiology (Munoz-Rubke et al., 2017; Mirdamadi et al., 2022) suggests such updates to body representation are communicated to the motor system to keep hand movements accurate. For example, even in the absence of motor learning, recalibration resulting from visuoproprioceptive cue conflict altered hand movements (Block and Liu, 2023). Perceptual recalibration has also been associated with changes in the primary motor cortex (M1) excitability. We used transcranial magnetic stimulation (TMS) to test M1 excitability before and after participants experienced conflicting or veridical visuoproprioceptive information about index finger position with no performance feedback (Munoz-Rubke et al., 2017). Results indicated that M1 excitability decreased in association with proprioceptive recalibration but increased in association with visual recalibration (Munoz-Rubke et al., 2017). These M1 changes were somatotopically focal, affecting only the index finger representation and not forearm or biceps (Mirdamadi et al., 2022).

**Figure 1.**
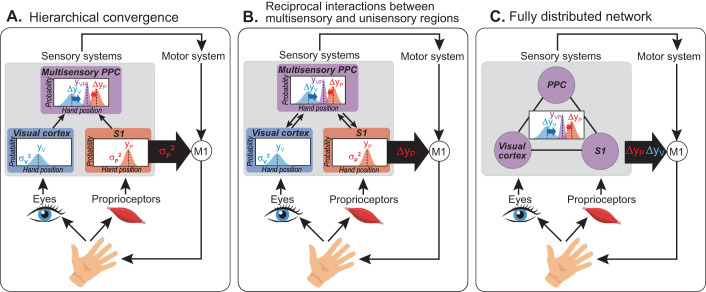
Three conceptual frameworks for how multisensory integration might be implemented in the brain, with implications for the role of the somatosensory cortex. These schematic diagrams are intended to illustrate alternative hypotheses about functional relationships. They are not intended to specify exact anatomical connections or computational mechanisms. ***A***, A hierarchical convergence model in which visual and proprioceptive signals about hand position are first processed in unisensory cortical regions: early visual areas and primary somatosensory cortex (S1), respectively. Signals from these regions would converge in multisensory parts of PPC, providing the visual and proprioceptive variance (*σ_V_*^2^, *σ_P_*^2^), and unimodal position estimates (*y*_V_, *y*_P_), needed for PPC to weight and integrate them [with weight of vision vs proprioception (w_V_) determined by various environmental and internal factors (Ghahramani et al., 1997; Ernst and Banks, 2002; Sober and Sabes, 2003)] and compute how much each unimodal estimate should recalibrate (Δ*y*_V_, Δ*y*_P_; blue and red arrows). The magnitudes of Δ*y*_P_ and Δ*y*_V_ are inversely related, with the lower-weighted modality usually recalibrating more (Ghahramani et al., 1997; Block et al., 2013; Munoz-Rubke et al., 2017). These multisensory updates of hand representation would be conveyed to the motor system only via PPC in this view. S1 might convey low-level proprioceptive processing (*σ_P_*^2^) to M1 (assessed by SAI in Expt. 1; thick black arrow) and cTBS over S1 might affect Δ*y*_P_ indirectly by increasing *σ_P_*^2^ (Expt. 2). ***B***, If visuoproprioceptive integration involves reciprocal interactions between unisensory and multisensory areas, then S1 activity might reflect some of the results of multisensory computations in PPC, such as proprioceptive recalibration (Δ*y*_P_). If so, we would expect cTBS over S1 to affect Δ*y*_P_ (Expt. 2). If it conveys this information to M1, changes in SAI should specifically reflect Δ*y*_P_ (Expt. 1). We would also expect the normal inverse relationship between Δ*y*_V_ and Δ*y*_P_ (thought to be mediated by PPC; Block et al., 2013) to be intact. ***C***, If visuoproprioceptive integration takes place in a fully distributed framework, then all regions in the network (PPC, S1, visual areas) could potentially reflect the results of multisensory computations. In this case, we would expect both visual and proprioceptive recalibration to be related to SAI (Expt. 1). We might also observe an effect of S1 cTBS on Δ*y*_V_, Δ*y*_P_, or both, and potentially a disruption of the normal inverse relationship between Δy*_V_* and Δy*_P_* (Expt. 2).

The neural mechanism conveying multisensory body representation updates to the motor system remains unknown. It likely depends on how the brain integrates and recalibrates visuoproprioceptive signals, a debated topic itself (Fig. 1). In a hierarchical model, recalibration would influence the motor system only via the posterior parietal cortex (PPC), after unisensory processing (Parra et al., 2022). However, early-stage multisensory convergence (Foxe and Schroeder, 2005), such as cross-modal modulation between S1 and V1 (Bieler et al., 2017) and reciprocal influences between unisensory and multisensory regions (Macaluso, 2006), suggests recalibration signals could also exist in S1 or V1. Finally, a fully distributed framework would posit that multisensory integration and recalibration emerge from a network without strict hierarchy (Ghazanfar and Schroeder, 2006; Chandrasekaran, 2017), allowing any involved region to convey recalibration to the motor system.

S1 is an intriguing candidate region to test these predictions, being linked both with multisensory parietal regions (Bolognini and Maravita, 2007; Filimon et al., 2009; Reichenbach et al., 2014; Isayama et al., 2019; Limanowski and Friston, 2020; De Vitis et al., 2022) and frontal motor regions, including M1. The degree to which somatosensory input affects motor output, termed sensorimotor integration, can be measured neurophysiologically: Short-latency afferent inhibition (SAI) describes the inhibitory effect of a peripheral electrical stimulus delivered before a motor response evoked by TMS (Tokimura et al., 2000). Inhibition is thought to be mediated at the cortical level by projections from S1 pyramidal cells to M1 interneurons (Cash et al., 2015; Turco et al., 2018).

We investigated whether S1 conveys hand representation updates to the motor system and whether it reflects multisensory computations or only unisensory processing. In Experiment 1 (Expt. 1), we measured SAI before and after participants estimated their left index finger's position using veridical or conflicting visuoproprioceptive cues. In Experiment 2 (Expt. 2), participants received continuous theta burst stimulation (cTBS) over S1, M1, or sham, estimating finger position before and after cTBS followed by visuoproprioceptive conflict to induce recalibration. Based on previously observed associations between M1 excitability and recalibration (Munoz-Rubke et al., 2017; Mirdamadi et al., 2022), the M1 group was included to test the specificity of any effects of S1 cTBS and to assess whether SAI measures could reflect changes in S1, M1, or both.

Because of S1's known role in unisensory proprioceptive processing, we expected its activity to be related to variability in proprioceptive estimates. Proprioceptive recalibration, however, stems from the multisensory comparison between visual and proprioceptive estimates (Ghahramani et al., 1997; Block et al., 2013; Munoz-Rubke et al., 2017). In a fully hierarchical convergence model, S1 activity would not reflect recalibration at all. Conversely, evidence of proprioceptive recalibration in S1 activity would be consistent with reciprocal interactions between S1 and PPC. Evidence of both visual and proprioceptive recalibration in S1 activity would support an even more distributed framework, where multisensory variables are represented throughout the network (Fig. 1).

## Materials and Methods

### Participants

A total of 121 self-reported right–handed healthy young adults participated in the study. All participants had normal or corrected-to-normal vision and no contraindications to TMS (Rossini et al., 2015). Participants self-reported that they were free of neurological, musculoskeletal, attention, or learning conditions. Procedures were approved by the Indiana University Institutional Review Board. All participants provided written informed consent before participating in the study.

### Perceptual alignment task general methods

Experiments 1 and 2 used variations of the same behavioral task to examine participants' perceived hand position using visual, proprioceptive, or bimodal cues. In addition, participants in both experiments were asked to rate their level of attention, fatigue, and pain from TMS at the conclusion of each session to evaluate whether different sessions or groups had similar subjective experiences. Participants were also questioned about their perceptions of the bimodal target to evaluate whether they perceived an offset during cue conflict. Details of the session and trial block design, which differ between experiments, are explained in their respective sections.

#### Apparatus (Fig. 2A)

Participants were seated in front of a custom 2-D virtual reality apparatus with a two-sided infrared touchscreen (PQ Labs) and a mirror positioned at the eye level. The touchscreen was positioned beneath the mirror such that visual information appeared in the plane of the touchscreen. A fabric drape attached to the apparatus was fastened around their neck, preventing vision of their limbs or surrounding environment. The participants always kept their right hand above the touchscreen and their left hand below the touchscreen.

**Figure 2.**
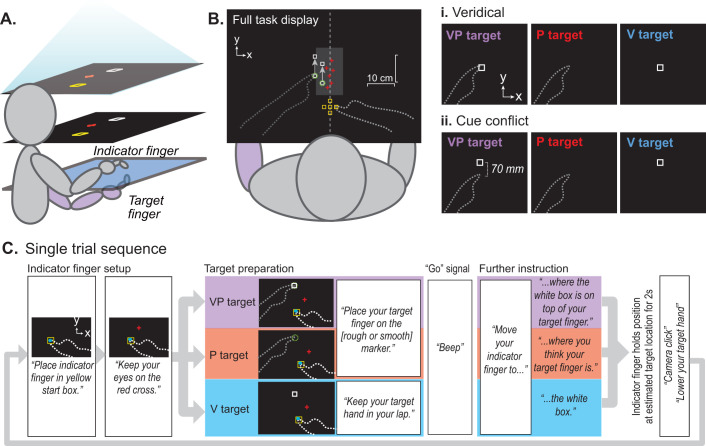
Visuoproprioceptive alignment task. ***A***, Apparatus. Participants viewed task display in a horizontal mirror (middle layer), making images appear to be in the plane of the touchscreen (bottom layer). Participants used their right index finger (indicator finger) on top of the touchscreen to indicate perceived target positions related to the left (target) index finger below the touchscreen. Participants had no direct vision of their hands at any time, and upper arms were covered with a drape. ***B***, Indicator finger starting positions varied randomly among five locations centered at the body midline (yellow squares). Targets varied between two positions slightly to the left of body midline (green circles), ∼30 cm in front of the body and well within reach. Participants were asked to gaze at a randomly positioned red cross within a zone along the midline rather than at any visual targets (white squares). ***i*–*ii*,** The three target types, presented in a pseudorandom order, were indicated by both visual and proprioceptive cues (VP target), proprioceptive cues only (P target), or visual cues only (V target). The target fingertip on a tactile marker beneath the touchscreen provided proprioceptive cues, and a white square provided visual cues. Not to scale. Dashed lines were not visible to subjects. No performance feedback was ever available. During cue conflict blocks (***ii***), the white square gradually shifted forward imperceptibly for both VP and V targets, 1.67 mm per trial, to a max of 70 mm. ***C***, Single-trial sequence. Each trial began with audio instructions for positioning the indicator finger, on top of the touchscreen, in the yellow start box. A blue cursor appeared during this phase, only until the indicator finger was correctly positioned. Instructions for positioning the target finger beneath the touchscreen came next, followed by the “go” signal. If the indicator finger did not leave the start box after 3 s, further audio instructions were played to remind the subject what to do. When the indicator finger had left the start position, touched down in a new position (without dragging), and held still for 2 s, the endpoint position was recorded, and a camera click sound played to let the subject know the trial was over.

#### Target types (Fig. 2B)

Participants were asked to use their unseen right index fingertip (indicator finger) to indicate the position of one of the three target types: a visual only target (V), a proprioceptive only target (P), and a visuoproprioceptive target (VP). For the V target, participants were asked to indicate their perceived position of a projected visual white square (1 cm). For the P target, participants were asked to indicate their perceived position of their left index fingertip, which was placed on one of two tactile markers below the touchscreen. For the VP target, participants were asked to indicate their perceived position of the white box projected on top of their left index fingertip. All subjects were told explicitly that the white box would always be right on top of their target fingertip on VP target trials. For V target trials, participants rested their left hand in their lap.

#### Single-trial procedure (Fig. 2C)

Each step of the trial was cued with recorded audio instructions. The trial began with the participant placing their indicator fingertip in a yellow square that served as the starting position. The starting position was presented at one of the five locations centered at the body midline, ∼20 cm in front of the chest. A blue cursor representing their indicator fingertip was initially shown to help position their fingertip in the starting square, which then disappeared to avoid any feedback cues. Next, participants were asked to fixate a red cross that appeared at random coordinates within a 10 cm zone along the body midline. They were asked to try to keep their gaze there throughout the remainder of the trial to reduce the chances that gaze behavior might differ between different target types. Participants were then instructed where to place their target hand (in the lap for V targets, on one of the tactile markers for P and VP targets). The tactile marker (1 cm) used in the P and VP targets was one of two positions (3 cm apart), resulting in 10 start position-target position pairs, requiring different directions and extents of movement by the indicator hand. The purpose of this was to make it less likely that participants would simply repeat the same motion with their indicator hand rather than trying to estimate the target position.

The proprioceptive cue of P and VP targets was achieved by having participants actively position their own left finger on the tactile marker. They had to lower their hand to their lap between each trial, and it remained in the lap for V trials. The purpose was to make sure that every P and VP trial involved the subject recently moving their own hand, which is thought to reduce proprioceptive drift (Wann and Ibrahim, 1992). Recent active movement may also improve proprioceptive salience, so that across participants, reliance on visual versus proprioceptive cues will be approximately even. In the present study and previous studies, this procedure results in people relying equally or slightly more on proprioception (Liu et al., 2018; Block and Sexton, 2020), enabling us to see the full range of possible recalibration behaviors (some people will recalibrate vision more than proprioception and vice versa).

When both hands were correctly positioned (as determined by the task program from touchscreen feedback), participants heard a go signal and, at their own pace, lifted their indicator finger off the touchscreen and placed it down where they perceived the target to be. If they hesitated longer than 3 s, further instructions played to remind them what to do (Fig. 2*C*). There was no speed requirement or knowledge of performance or results. Adjustments were permitted, and the trial terminated after the indicator fingertip remained still on the touchscreen for 2 s. The *x*–*y* coordinates of the indicator finger endpoint served as a proxy for where the participant perceived the target to be.

### Expt. 1 design

#### Participants

**Figure 3.**
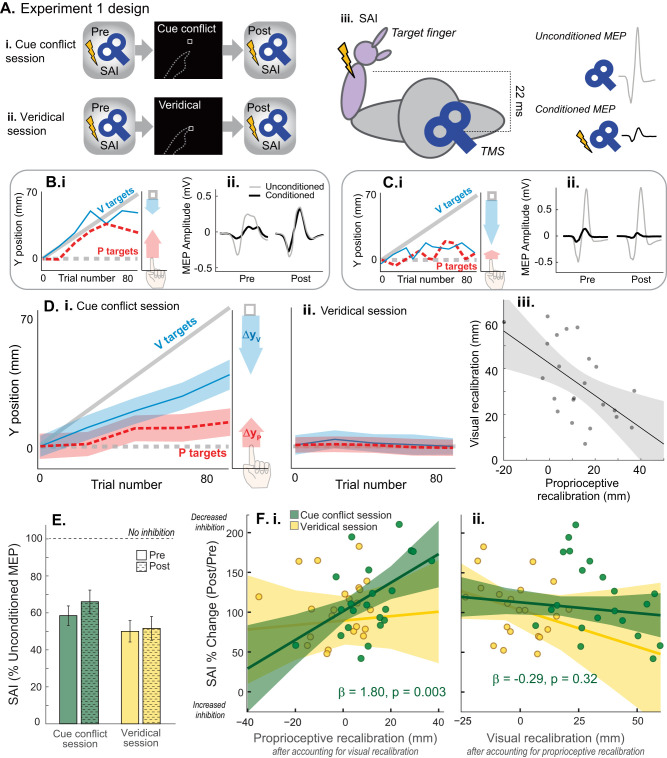
Expt. 1 design and results. ***A***, Expt. 1 design. All participants completed two sessions in a random order. SAI was assessed immediately before and after the behavioral portion of each session. ***i***, Cue conflict task consisted of V, P, and VP trials, with the visual cue gradually shifting forward relative to the proprioceptive cue. ***ii***, The veridical session included the same number of V, P, and VP trials, with cues displayed veridically throughout. ***iii***, SAI was assessed by delivering an electrical stimulus to the left median nerve 22 ms prior to a suprathreshold TMS pulse over right M1. Top, Schematic unconditioned MEP. Bottom, Schematic of MEP conditioned by median nerve stimulus, illustrating inhibition of motor output by somatosensory input. ***B*, *C***, Expt. 1 example participants' performance on P and V targets in the cue conflict block (averaged every four trials for clarity; ***i***) and their corresponding changes in SAI in the cue conflict session (***ii***). Undershooting the visual target on V trials represents visual recalibration (blue arrow), and overshooting the proprioceptive target on P trials represents proprioceptive recalibration (red arrow; ***i***). ***B***, Participant with relatively high proprioceptive recalibration (***i***) demonstrated a decrease in SAI (larger ratio of conditioned relative to unconditioned MEP) post-task relative to pre (***ii***). ***C***, Participant with relatively larger visual recalibration demonstrated an increase in SAI (smaller ratio of conditioned relative to unconditioned MEP) post-task compared with pre. ***D***, Group means on unimodal P and V trials (red and blue) during the cue conflict session (***i***) and veridical session (***ii***), averaged every four trials for clarity. Shaded regions represent SEM. ***iii***, In the cue conflict session, individuals who recalibrated proprioception to a greater extent recalibrated vision to a lesser extent (*r* = −0.52; *p* = 0.013). ***E***, Group mean SAI pre- and post-alignment task for the cue conflict and veridical sessions. SAI was expressed as the conditioned MEP amplitude relative to the unconditioned MEP amplitude (TS alone). Values <100 indicate inhibition, with smaller values denoting greater inhibition. Error bars indicate standard error of the mean. SAI did not differ significantly between sessions or timepoints. ***F***, ***i*****–*ii***, Change in SAI (post divided by pre) plotted against predictor residuals, with lines of best fit and corresponding 95% CIs. In the cue conflict session, recalibration of either modality in the positive direction is beneficial (helps compensate for the visuoproprioceptive mismatch). ***i***, After statistically controlling for the effect of visual recalibration, large proprioceptive recalibration in the cue conflict session (green) was significantly associated with reduced SAI (disinhibition). There were no associations between proprioceptive recalibration in the veridical session (yellow) and SAI. ***ii***, After controlling for the effect of proprioceptive recalibration, there were no significant associations between visual recalibration and change in SAI for either session.

Twenty-two adults (21.6 ± 3.68 years, mean ± SD; 12 males) participated in Expt. 1. Participants completed two sessions each. In the cue conflict session, a 70 mm mismatch between visual and proprioceptive cues about the left index fingertip was gradually imposed in the sagittal plane. In the veridical session, visual and proprioceptive information remained veridical. The sessions were separated by at least 5 d, with order randomized, to minimize carry-over effects. On average there were 16 ± 14 d (mean ± SD) between sessions. TMS measurements were performed pre- and post-perceptual alignment task in each session (Fig. 3*A*).

#### Behavior

##### Perceptual alignment task blocks

There were two blocks of trials in each session. The first block was identical between sessions and consisted of a baseline veridical block with 15 V, 15 P, and 10 VP trials that were pseudorandomized. The second block comprised 84 trials (42 VP, 21 V, and 21 P, alternating order). In the cue conflict session (Fig. 3*Ai*), the white box was gradually shifted forward from the target index fingertip on VP trials in the second block. The offset increased by 1.67 mm per VP trial. By the end of the block, the visual cue was displaced 70 mm forward of the proprioceptive cue, in the sagittal plane. Participants rarely become aware of this manipulation (Hsiao et al., 2022). In the veridical session, there was no offset, and VP targets remained veridical throughout (Fig. 3*Aii*).

##### Visual and proprioceptive recalibration

Figure 3, *Bi* and *Ci*, shows the indicator finger estimates relative to the true positions of V and P targets for two exemplar participants across trials in the cue conflict block. Similar to past work, the magnitude of visual and proprioceptive recalibration (Δ*y*_P_ and Δ*y*_V_) was calculated using indicator fingertip endpoints of the V and P targets, respectively. The average *y*-estimate of the first four trials of the given modality was subtracted from the average *y*-estimate of the last four:

$$\Delta y_{p}\;=\;\mathrm{last}\;4\;P\;\mathrm{endpoints}\;\text{–}\;\mathrm{first}\;4\;P\;\mathrm{endpoints},\;(1)$$

$$\Delta y_{V}\;=\;70\;\text{–}\;(\mathrm{last}\;4\;V\;\mathrm{endpoints}\;\text{–}\;\mathrm{first}\;4\;V\;\mathrm{endpoints}).\;(2)$$

Recalibration was expressed relative to the change in true *y*-position of the target (70 mm for V targets; 0 mm for P targets at the end of the cue conflict block; 0 mm for both modalities in a veridical block). In the cue conflict condition, a positive recalibration value indicates recalibration in the expected direction (i.e., undershooting for V targets and overshooting for P targets).

##### Weighting

Weighting was calculated using the 2D endpoint indicator fingertip position on VP targets relative to the unimodal targets during the first block of each session (veridical targets). For instance, a person that relies more on vision would be expected to have their mean estimate of VP targets closer to the mean estimate of V targets compared with P targets. Based on the formula in Equation (3), a weighting value >0.5 indicates greater reliance on vision, whereas a value <0.5 indicates greater reliance on proprioception:

$$W_{v}\;=\;\;\frac{|y_{P}-\;y_{VP}|}{\left(|y_{P}-\;y_{VP}|)+(|y_{V}-\;y_{VP}|\right)}.\;(3)$$

Consistent with previous work (Block and Bastian, 2010), we computed a separate *W*_v_ for each VP trial, comparing the VP trial endpoint with the four closest V and four closest P trials in the sequence. If the V and P trial endpoints were too closely overlapping (<0.5 SD apart), no *W*_v_ was computed for that VP trial.

##### Visual and proprioceptive endpoint variance

Variance was computed for the 2D indicator finger endpoints on unimodal V and P trials during the first block of each session (veridical). This was accomplished by converting each endpoint to a vector relative to the mean [*x*, *y*] coordinates of the cluster of endpoints. Variance was then computed for the vector magnitudes. To approximate the variance of participants' visual and proprioceptive estimates (as opposed to the variance of the endpoints, which includes motor and proprioceptive variance from the indicator hand), we subtracted 0.5 P variance from each estimate (Block and Bastian, 2010). This assumes that the variance contributed by the indicator hand to variance of any target type is half of the variance apparent in the indicator finger endpoints on unimodal P targets, which of course is an approximation.

#### Neurophysiology

##### M1 stimulation

TMS was applied over the M1 hand representation in the right hemisphere to probe neurophysiology pertaining to the target hand that experiences visuoproprioceptive cue conflict. Single monophasic TMS pulses were delivered using a Magstim 200^2^ stimulator (Magstim) with a 70 mm figure-of-eight coil. The coil was held tangentially to the scalp with the handle 45° posterolateral from the midline to elicit posterior-to-anterior current in the right M1. The hotspot was identified by the scalp position that elicited the largest and most consistent response in the left first dorsal interosseous (FDI) muscle. The location and trajectory were registered in BrainSight neuronavigation system (Rogue Research) for consistent coil positioning throughout the session. Surface electromyography (EMG) was recorded from the left FDI muscle and abductor pollicis brevis (APB) muscle using a belly–tendon montage and a ground electrode over the ulnar styloid process. EMG recordings were amplified (AMT-8; Bortec Biomedical), bandpass filtered (10–1,000 Hz), sampled at 5,000 Hz, and recorded using the Signal software (Cambridge Electronic Design). At the beginning of each session, we found resting motor threshold (RMT), defined as the minimum intensity that elicits a twitch ≥50 µV in at least 10 out of 20 trials (Rossini et al., 2015). We then found the test stimulus (TS) intensity that elicited a twitch of 1 mV on average over 10 trials.

##### SAI procedure

SAI was assessed before and after the perceptual alignment task in each session (Fig. 3*A*). To elicit SAI, a TMS pulse at TS intensity was delivered 22 ms after an electrical stimulus at the left median nerve. Electrical stimuli were delivered with a Grass Instruments S88 stimulator (Astro-Med) with an in-series stimulus isolation unit (SIU-5) and a constant-current unit (CCU-1; square wave pulse, 0.2 ms duration, cathode proximal). The intensity was set based on the lowest intensity that elicited a slight thumb twitch and consistent APB M-wave amplitude. The M-wave amplitude was monitored online and kept constant throughout the session (Turco et al., 2018; Mirdamadi and Block, 2020).

Twenty conditioned pulses (median nerve stimulus + TS) and 20 unconditioned pulses (TS alone) were delivered in a random order, with an interstimulus interval of ∼ 5 s, both pre- and post-alignment task. We adjusted the TS intensity post-alignment task, if needed, to elicit the same size unconditioned MEP response of ∼1 mV. Therefore, any changes in SAI reflect changes in somatosensory projections to M1 rather than changes in M1 excitability alone.

SAI magnitude was expressed as a percentage of the average conditioned MEP peak-to-peak amplitude relative to the unconditioned peak-to-peak amplitude (Fig. 3*Aiii*). Therefore, lower values of SAI within a time point (pre or post) indicate greater inhibition by the somatosensory afferent volley. We also calculated the change in SAI (post/pre) for each session. Smaller delta SAI indicates greater inhibition post relative to pre, while larger delta SAI values indicate less SAI post relative to pre.

#### Statistical analysis

Baseline (pre-alignment task) neurophysiology was compared between the veridical and cue conflict sessions using paired *t* tests for RMT, TS MEP amplitude, and SAI. To test whether the alignment task influenced SAI differently across sessions, we performed a session (cue conflict vs veridical) × time (pre vs post) repeated-measure ANOVA.

Multilevel hierarchical modeling was used to assess whether an individual's magnitude of proprioceptive or visual recalibration was related to their change in SAI. By including both visual and proprioceptive recalibration as predictors in the regression model, we can examine the association between proprioceptive recalibration and change in SAI after the variance explained by visual recalibration is accounted for and vice versa. Similarly, when assessing the relationship between visual recalibration and change in SAI, the variance explained by proprioceptive recalibration is accounted for.

We first examined a full model that included predictor variables for the interaction between session and modality and their main effects. However, the full model had variance inflation factor (VIF) values that suggested the presence of multicollinearity (VIF value of 6). Therefore, we computed a reduced model that only had the interaction term of session and modality, which had VIF values that ranged between 1.04 and 1.05. Since the reduced model had less multicollinearity and was not statistically different from the full model (*χ*^2^ test: *p* = 0.7), the reduced model was used for analysis.

To illustrate the results of this model in Figure 3*F*, we used predictor residual plots in which the *x*-axis represents the residuals of the predictor of interest (e.g., proprioceptive recalibration) after regressing out the effect of the other predictor (e.g., visual recalibration). This approach isolates the unique variance of the predictor of interest, ensuring that the plotted relationship with SAI reflects its independent contribution, free from the influence of the other recalibration modality. We were also interested in whether proprioceptive target estimation variance was related to the change in SAI. We therefore repeated the multilevel hierarchical model with proprioceptive and visual variance instead of recalibration.

Finally, we computed Pearson's correlation coefficients between baseline SAI (pre-alignment task) and 2D *W*_v_ in the veridical session, and between baseline SAI and P variance and V variance in the veridical session. We also computed the correlation between baseline SAI and proprioceptive recalibration in the cue conflict session. *α* was set at 0.05.

### Expt. 2 design

#### Participants

Eighty-one individuals (21.68 ± 4.32 years, mean ± SD; 38 male) participated in Expt. 2. All were right-handed according to the Edinburgh Handedness Inventory (Oldfield, 1971). Participants were assigned to one of three stimulation groups: S1, M1, or Sham (*N* = 27 per group). Group assignment was random, following a block randomization table. Group assignment was revealed to the experimenters only when it was time to deliver cTBS, after task training and baseline measures were complete. S1 had 12 male participants (21.44 ± 3.94 years, mean ± SD), M1 had 13 male participants (21.3 ± 4.58 years, mean ± SD), and the Sham group had 13 male participants (22.3 ± 4.28 years, mean ± SD). The sample size was determined a priori with a power analysis on pilot data in earlier participants who received S1, M1, or no cTBS (*N* = 8, 13, 27) with the same session design. We determined a total sample size of 81 would be needed to have 80% power to detect an effect size *f* of 0.404 with *α* of 0.05 with a one-way ANOVA on three groups. The effect size was determined for proprioceptive recalibration in the pilot subjects.

**Figure 4.**
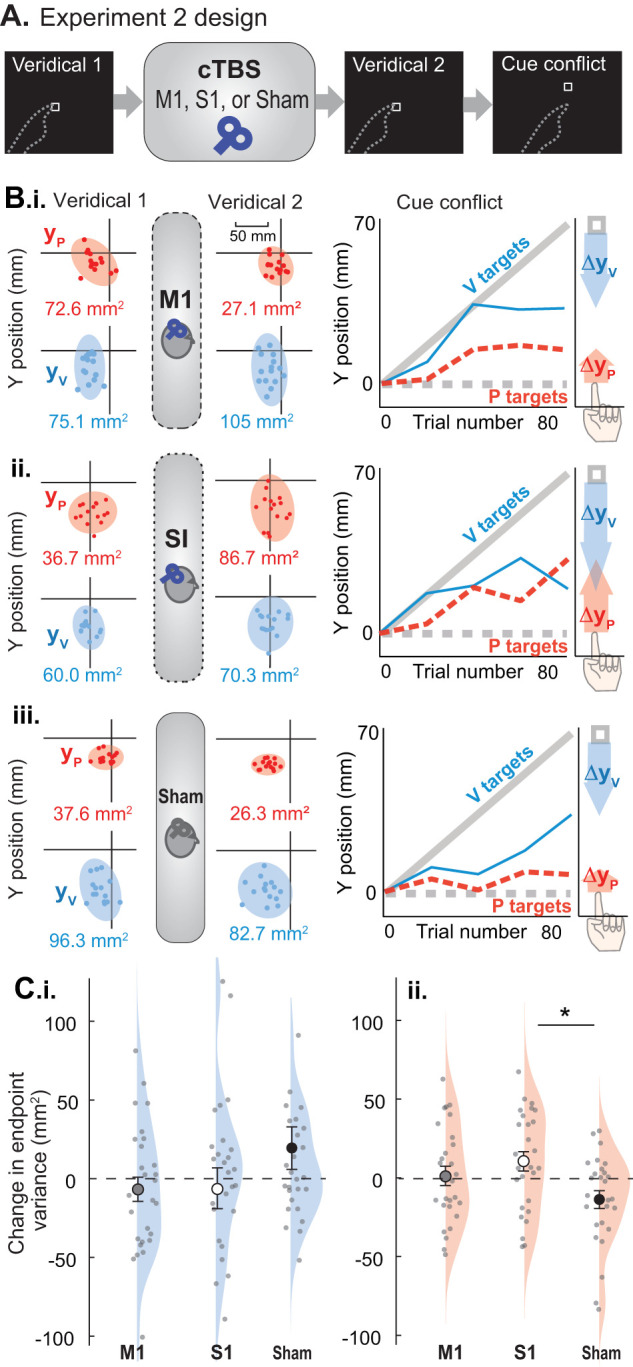
***A***, Expt. 2 design. After a baseline block of V, P, and veridical VP trials, participants received M1, S1, or sham cTBS according to random group assignment. After a second block of veridical trials, participants performed a cue conflict block in which the visual cue gradually shifted forward relative to the proprioceptive cue. ***B*,** ***i*****–*iii***, **Three example participants’ performance in the three blocks**. Indicator finger endpoints in 2D on P (red) and V (blue) unimodal trials before and after cTBS (Veridical 1 and 2). One example participant is shown for each group. Data plotted with target always at the origin. Each participant was seated in the direction of the negative *y*-axis. Shaded ellipses represent 95% confidence intervals, whereas red and blue numbers represent 2D variance calculated as described in Materials and Methods. Each person's performance during the cue conflict block is also displayed, with shaded arrows representing recalibration in visual (blue) and proprioceptive (red) estimates. Group recalibration results are presented in Figure 5. ***C***, **Group mean changes and standard errors on unimodal V (*i*) and P trials (*ii*) from Veridical 1 to Veridical 2**. Dots represent individual participants. ***i***, There was no effect of group on change in visual variance. Not pictured: one outlier in the sham group at 332 mm^2^ and one outlier in the S1 group at −243 mm^2^. ***ii***, S1 cTBS increased proprioceptive variance relative to sham cTBS. All participants are visible in this axis range.

cTBS was delivered in between two veridical perceptual alignment blocks (Fig. 4*A*) to assess any effect of cTBS on baseline behavior. This was followed by a cue conflict block. The second veridical block and the cue conflict block took ∼45 min for most subjects to complete, such that all subjects finished the behavioral tasks within an hour of receiving cTBS.

#### Behavior

The apparatus, target types, and task instructions were identical to that of Expt. 1. Each participant performed three blocks of perceptual alignment task trials: Veridical 1, Veridical 2, and a cue conflict block, with cTBS delivered after Veridical 1 (Fig. 4*A*). Each veridical block consisted of 40 trials in repeating order (15V, 15 P, 10 VP). After the second veridical block, participants completed the same cue conflict block used in Expt. 1. Recalibration and weighting were computed as in Expt. 1. Endpoint variance was computed as in Expt. 1, except we did not subtract 0.5 P target variance from each variance estimate because the planned analyses were a pre–post design.

In order to test whether cTBS over S1 had any effect on tactile sensitivity that could interfere with participants' ability to feel the tactile markers with their target finger, we performed an abbreviated grating orientation test (GOT) before and after cTBS for all participants. That is, the first GOT was performed after Veridical 1 and before cTBS, and the second GOT was performed after cTBS and before Veridical 2. We used J.V.P. DOMES for cutaneous spatial resolution measurements. The set consists of domes of 35, 0.5, 0.75, 1.0, 1.2, 1.5, 2, 3, 4, and 5 mm grating width. For the assessment, participants had their eyes closed and their left hand resting on the table with the palm facing up. They were tested on their left index fingertip palmar surface. The domes were attached to a force gauge to maintain uniformity in the pressure (0.65–0.95 N) applied while testing based on an earlier study (Van Boven and Johnson, 1994). The testing started with a 3 mm grating width and a random order of orientation (vertical or horizontal) was used. No feedback was provided to the participant. If the subject correctly reported six orientations consecutively, then a grating of smaller width was used next. If they reported one or more orientations incorrectly, then a larger grating was used. This continued until they were unable to report the orientation correctly. The smallest grating width that participants reported correctly was noted as their tactile sensitivity threshold.

#### TMS

TMS was delivered using a using a Magstim Super Rapid Plus stimulator with a D70^2^ 70 mm figure-of-eight coil (Magstim). Single pulses were first delivered over the right M1 to find the RMT of the left FDI muscle using identical methods as Expt. 1. Brainsight Neuronavigation was used as in Expt. 1, with participants' heads registered to the template brain for consistent coil positioning throughout the session.

cTBS was delivered over the right M1 or right S1 to modulate the hemisphere that pertains to the left hand (target hand), which experiences the visuoproprioceptive cue conflict. For the M1 group, cTBS was delivered over the FDI motor hotspot. The S1 stimulation target was defined 2 cm lateral and 1 cm posterior to the M1 target (Holmes and Tamè, 2018; Holmes et al., 2019; Mirdamadi and Block, 2021). Given the limitations on depth of stimulation in a flat coil (Deng et al., 2013), regions within S1 most likely stimulated are Areas 1, part of 2, and part of 3b; Area 3a is likely too deep in the central sulcus to be stimulated effectively with this method (Siebner et al., 2022). The sham target was the same as the M1 target but consisted of an unplugged coil placed on the scalp. A second coil, held behind the participant's head out of their view, was plugged in so that the sounds of the cTBS train would be audible.

cTBS was delivered at 70% of RMT (Gentner et al., 2008; Goldsworthy et al., 2014; Mirdamadi and Block, 2021), consisting of triplets of 50 Hz repeated at 5 Hz for 40 s (Huang et al., 2005). Participants sat quietly for 5 min before and after cTBS to avoid any potential reversal of the after-effects (Iezzi et al., 2008).

#### Statistical analysis

To determine whether cTBS affected endpoint variance on unimodal P and V targets, we subtracted individual values for Veridical 2 minus Veridical 1 and ran a one-way ANOVA on the difference across groups. We analyzed weighting of vision versus proprioception the same way (*W*_v_). Two participants had closely overlapping distributions of P and V endpoints in one of the veridical blocks, and insufficient *W*_v_ were calculated to form an estimate for the whole block. These participants were excluded from the *W*_v_ analysis. ANOVAs were followed by Tukey's HSD post hoc comparisons upon significant result. Each distribution was checked for normality and equality of variances (Shapiro–Wilk test and Levene's test, respectively). Only the change in V target variance violated any assumptions (due to an outlier in the Sham and S1 groups), so the nonparametric equivalent (Kruskal–Wallis) was used instead.

We tested for group effects on P, V, and total recalibration (sum of P and V recalibration, to approximate the total proportion of the 70 mm conflict participants compensated for) using separate one-way ANOVAs, which were followed by Tukey's HSD post hoc comparisons upon significant result. GOT thresholds pre-cTBS were subtracted from post-cTBS, and these changes were compared across groups using the Kruskal–Wallis test. *α* was defined as 0.05 for all hypothesis tests.

### Control experiment design

#### Participants

Eighteen individuals (4 males; 22.22 ± 4.41 years, mean ± SD) participated in the control experiment. All were right-handed according to the Edinburgh Handedness Inventory (Oldfield, 1971).

#### Behavior

Because the main experiments used the same method to introduce a cue conflict—forward displacement of the visual target from the proprioceptive target—there is the potential for a confound related to visual recalibration. During cue conflict blocks, the white square shifted forward on both VP and V targets, the latter of which were used to assess visual recalibration. Ideally, participants should place their indicator finger at the perceived location of the white square on V trials, whether shifted or not. If they undershoot by the end of the cue conflict block relative to the beginning of the block, we infer that they perceive the visual target to be closer than it actually is (visual recalibration). The potential confound is that participants might have a general tendency to avoid moving their indicator hand farther away, i.e., effort minimization. This could result in undershooting the visual target as it gets farther away.

To control for this possibility, participants performed a behavioral experiment identical to the perceptual alignment task in Expt. 2, except that the “cue conflict” block consisted only of V trials. The white box shifted forward at the same rate as the main experiments, reaching 70 mm forward displacement after 84 trials. Participants were trained on all three target types as in the main experiments, but there were no P or VP trials during the V target shift, so there was no cue conflict. Thus, we did not expect visual recalibration (undershoot of V targets) unless participants were minimizing movement distance of the indicator hand. Visual recalibration was calculated the same as the main experiments (Eq. 2).

#### Statistical analysis

We tested whether visual recalibration differed significantly from zero using a one-sample *t* test. For context, we applied the same test to visual recalibration in the cue conflict and veridical sessions of Expt. 1 (expected to be greater than zero and similar to zero, respectively) and in Expt. 2 sham group (expected to be greater than zero). In each case, the test was performed two-tailed with *α* of 0.05. Cohen's *d* was computed for effect size.

##### Code accessibility

The data and analysis code used to produce this manuscript are publicly available at https://osf.io/w5pfk/.

## Results

### Expt. 1

#### Behavior

Each participant completed both a cue conflict session and a veridical session, on different days in a random order (Fig. 3*A*). In the cue conflict session, the visual cue was gradually shifted forward of the proprioceptive cue over the course of 84 trials, to a max of 70 mm in the *y*-dimension. On average, in the *y*-dimension, participants recalibrated vision 34.7 ± 6.9 mm (mean ± 95% CI) and proprioception 11.1 ± 5.3 mm (Fig. 3*Di*). Some participants recalibrated proprioception more than vision (Fig. 3*Bi*), while others recalibrated vision more than proprioception (Fig. 3*Ci*). As shown previously, visual and proprioceptive recalibration were inversely associated (*r* = −0.52; *p* = 0.013; Fig. 3*Diii*; Munoz-Rubke et al., 2017).

The *y*-dimension was of primary interest because that was the dimension of the cue conflict. However, to confirm the specificity of *y*-dimension recalibration, we also computed *x*-dimension recalibration in the cue conflict session. Participants recalibrated vision in the *x*-dimension −0.61 ± 5.7 mm (mean ± 95% CI) and proprioception −0.45 ± 6.8 mm (negative values represent a shift to the subject's left). This is consistent with recalibration in the *y*-dimension being a specific response to the cue conflict in the *y*-dimension. In the veridical session, there was no cross-sensory mismatch, so the amount of visual and proprioceptive recalibration is expected to be roughly zero on average. In line with previous work, in the *y*-dimension, participants recalibrated vision −1.2 ± 5.0 mm and proprioception −2.2 ± 4.9 mm (Fig. 3*Dii*; Munoz-Rubke et al., 2017; Mirdamadi et al., 2022).

#### Neurophysiology

We found no evidence that baseline neurophysiological measures differed across sessions (Table 1) or that SAI changed from pre-alignment task to post-alignment task timepoint in either session (ANOVA session × timepoint, *F*_(1,21)_ = 1.08; *p* = 0.31; session, *F*_(1,21)_ = 3.70; *p* = 0.068; timepoint, *F*_(1,21)_ = 2.29; *p* = 0.15; Fig. 3*E*). We also found no change over time in unconditioned motor-evoked potential (MEP) amplitude (session × timepoint, *F*_(1,21)_ = 0.31; *p* = 0.58; session, *F*_(1,21)_ = 0.08; *p* = 0.78; timepoint, *F*_(1,21)_ = 2.72; *p* = 0.11), indicating that any associations between SAI and recalibration behavior were not confounded by differences in motor cortex excitability.

**Table 1.** Expt. 1 summary of neurophysiological measures recorded at baseline in each session and subjective ratings recorded at the end of each session

	Cue conflict session (mean ± 95% CI)	Veridical session (mean ± 95% CI)
RMT (% MSO)	38.5 ± 2.5%	38.5 ± 3.0%
MEP at TS (mV)	1.0 ± 0.2 mV	1.0 ± 0.1 mV
SAI (% of unconditioned MEP)	58.5 ± 10.4%	50.0 ± 11.3%
Attention rating	7.5 ± 0.4	8.0 ± 0.3
Fatigue rating	4.4 ± 0.7	3.4 ± 0.8
Sleep rating	7.7 ± 0.5	7.4 ± 0.5
Pain rating	2.6 ± 0.8	2.8 ± 0.7

None differed across sessions (all *p* > 0.2).

RMT, resting motor threshold; MSO, maximum stimulator output; MEP at TS, MEP achieved at a TS intensity intended to elicit a 1 mV MEP; SAI, short-latency afferent inhibition. All ratings were on a scale from 1 to 10, reported verbally, with 10 being the best attention and sleep and the worst fatigue and pain.

Two example participants with different magnitudes of proprioceptive recalibration in the cue conflict session are shown in Figure 3. The individual with relatively larger proprioceptive recalibration had a decrease in SAI post-cue conflict (Fig. 3*Bii*), whereas the individual with smaller proprioceptive recalibration had an increase in SAI post-cue conflict (Fig. 3*Cii*). Consistent with this pattern, multilevel regression model results suggest that change in SAI from before to after the alignment task (post divided by pre) showed modality-specific recalibration associations with the cue conflict session, but not the veridical session (Table 2). These associations are illustrated with predictor residual plots (Fig. 3*F*), which allows us to show the relationship of one predictor variable with the dependent variable, after statistically controlling for the effect of the other predictor (McElreath, 2015). For either modality, recalibration in the positive direction is beneficial in the cue conflict session (helps compensate for the visuoproprioceptive mismatch).

**Table 2.** Expt. 1 multilevel regression results for change in SAI, comprising four interaction terms for session type (veridical or cue conflict) and recalibration modality (proprioceptive or visual)

Predictors	*β* (CI)	*t*_(39)_	*p*
*Fixed parts*			
Intercept	105.94 (89.63, 122.25)	13.14	**<0.001**
Veridical session: proprioceptive recalibration	0.28 (−1.20, 1.75)	0.38	0.704
Cue conflict session: proprioceptive recalibration	1.80 (0.63, 2.96)	3.12	**0.003**
Veridical session: visual recalibration	−0.99 (−2.46, 0.47)	−1.37	0.179
Cue conflict session: visual recalibration	−0.29 (−0.87, 0.30)	−1.00	0.324
*Random parts*			
NID	22		
Observations	44		

Columns represent the percentage of the baseline (post divided by pre) in SAI. *β*s are presented with their 95% CIs. The test statistic (*t*) is calculated from the parameter estimate divided by SE, and *p* values are calculated based on the *t* statistic with corresponding degrees of freedom. Boldface identifies statistically significant results.

After controlling for the effect of visual recalibration, greater positive proprioceptive recalibration was associated with greater decrease in SAI (less inhibition post-alignment task) in the cue conflict session (*β* = 1.80; *t*_(39)_ = 3.12; *p* = 0.003), but not the veridical session (*β* = 0.28; *t*_(39)_ = 0.38; *p* = 0.704; Fig. 3*Fi*). After controlling for the effect of proprioceptive recalibration, change in SAI was not significantly associated with visual recalibration (cue conflict session, *β* = −0.29; *t*_(39)_ = −1.00; *p* = 0.324; veridical session, *β* = −0.99; *t*_(39)_ = −1.37; *p* = 0.179; Fig. 3*Fii*).

Repeating the multilevel regression model analysis using visual and proprioceptive estimation variance (rather than recalibration) found that change in SAI from before to after the alignment task (post divided by pre) was not associated with the predictor of interest (proprioceptive variance in the cue conflict session, *β* = 0.17; *t*_(39)_ = 1.02; *p* = 0.32; 95% CI [−0.16–0.49]). This null result had moderately elevated collinearity (VIF = 4.19), which can inflate the standard error. However, even if truly significant, this *β* would be relatively small compared with the corresponding predictor in the recalibration model (proprioceptive recalibration in the cue conflict session; Table 2). This is consistent with change in SAI being related to proprioceptive recalibration specifically, rather than to proprioceptive variance. None of the other variance predictors were significantly associated with change in SAI (all *p* > 0.16).

In the veridical session, SAI at the baseline was negatively correlated with proprioceptive variance (*r* = −0.45; *p* = 0.034). Baseline SAI was not significantly correlated with visual variance (*r* = −0.11; *p* = 0.64) or with participants' weighting of vision versus proprioception on bimodal trials (*r* = 0.18; *p* = 0.42). In the cue conflict session, SAI at the baseline was not significantly correlated with proprioceptive recalibration (*r* = −0.19; *p* = 0.40).

### Expt. 2

#### Veridical blocks

Three groups of 27 participants performed a block of V, P, and veridical VP trials before and after cTBS was delivered over their target hand representation in S1, M1, or sham (Fig. 4*A*). We computed 2D variance of participants’ estimation endpoints on unimodal V and P trials in each veridical block to assess whether visual or proprioceptive variance changed after cTBS (Fig. 4*B*). Proprioceptive variance was similar across groups before cTBS (M1: 56.1 ± 16.6; S1: 53.8 ± 12.7; Sham: 60.5 ± 14.4 mm^2^; mean ± 95% CI). Proprioceptive variance changed differently across groups post-cTBS relative to pre, with the difference between pre and post showing an effect of group (ANOVA: *F*_2,78_ = 4.31, *p* = 0.017, *η*^2^ = 0.10). This was driven by an increase in proprioceptive variance post S1 cTBS relative to sham (*p* = 0.013; Fig. 4*Ci*). Visual variance was similar across groups before cTBS (M1, 72.2 ± 17.0; S1, 91.6 ± 30.7; sham, 68.5 ± 28.9 mm^2^; mean ± 95% CI). cTBS had no impact on visual target estimation variance regardless of the group (*χ*^2^ = 2.08; *p* = 0.35; Fig. 4*Cii*). This was also the case when the analysis was performed without the two outliers (*p* > 0.2).

We also computed an estimate of how much people were relying on vision versus proprioception when both were available (weighting), taking advantage of subjects’ naturally different spatial biases when estimating V, P, and VP targets (Foley and Held, 1972; Crowe et al., 1987), even with no cue conflict (Smeets et al., 2006). As computed, this parameter ranges from 0 (only proprioception) to 1 (only vision), with 0.5 indicating equal reliance on vision and proprioception. Weighting was similar across groups before cTBS, with values in line with past work (Liu et al., 2018; Block and Sexton, 2020; M1, 0.39 ± 0.07; S1, 0.46 ± 0.09; sham, 0.41 ± 0.09; mean ± 95% CI). cTBS had no impact on weighting regardless of group (*F*_(2,76)_ = 0.308; *p* = 0.74; *η*^2^ = 0.008).

#### Cue conflict block

**Figure 5.**
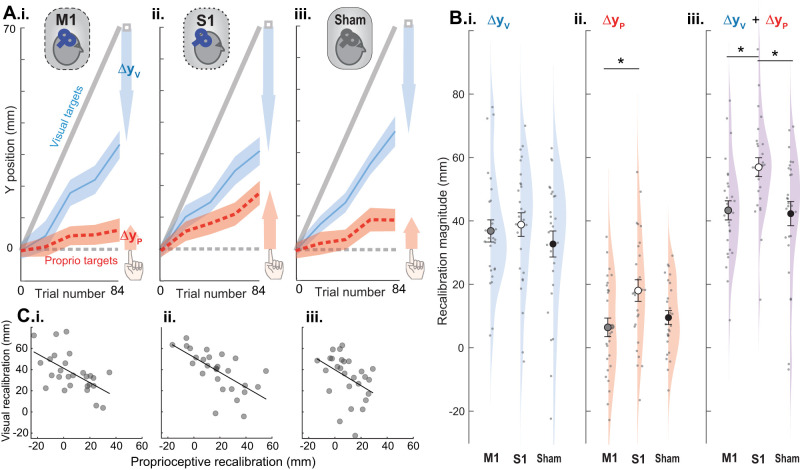
Expt. 2 group behavior during the cue conflict block. ***A***. ***i*–*iii***, Mean performance on P and V targets in the cue conflict block after M1, S1, and sham cTBS, respectively (averaged every 4 trials for clarity). Undershooting the visual target on V trials represents visual recalibration (blue arrow) and overshooting the proprioceptive target on P trials represents proprioceptive recalibration (red arrow). S1 participants recalibrated proprioception noticeably more than the other groups. Shading indicates SEM. ***B***, Comparing recalibration across groups. ***i***, Visual recalibration did not differ significantly across groups. ***ii***, Proprioceptive recalibration was larger in the S1 group compared with the M1 group. ***iii***, Total recalibration (sum of visual plus proprioceptive recalibration) for S1 was greater than M1 or sham groups. ***C****. **i-iii***, Across all groups, proprioceptive recalibration was negatively associated with visual recalibration.

On average, visual recalibration was 36.8 ± 7.2 mm for M1, 38.8 ± 7.7 mm for S1, and 32.7 ± 8.4 mm for sham (mean ± 95% CI). Proprioceptive recalibration was 6.5 ± 6.0 cm for M1, 18.1 ± 7.0 cm for S1, and 9.6 ± 4.5 mm for sham (Fig. 5*A*,*B*). Proprioceptive and visual recalibration were inversely correlated for all groups; individuals with larger proprioceptive recalibration had smaller visual recalibration and vice versa (M1, *r* = −0.57; *p* = 0.002; S1, *r* = −0.65; *p* < 0.001; sham, *r* = −0.40; *p* = 0.04; Fig. 5*C*). Across all three groups, proprioceptive estimation variance in the second veridical block had little effect on proprioceptive recalibration (*R*^2^ = 0.058), consistent with recalibration arising from multisensory computations involving more than just proprioception.

While visual recalibration was similar across groups (*F*_(2_,_78)_ = 0.68; *p* = 0.51; *η*_P_^2^ = 0.017; Fig. 5*Bi*), the magnitude of proprioceptive recalibration (Fig. 5*Bii*) and total recalibration (Fig. 5*Biii*) differed across groups (*F*_(2_,_78)_ = 4.35; *p* = 0.016; *η*_P_^2^ = 0.100; *F*_(2_,_78)_ = 6.18; *p* = 0.003; *η*_P_^2^ = 0.137). Proprioceptive recalibration was larger following S1 cTBS relative to M1 cTBS (*p* = 0.015) but not sham (*p* = 0.099). In contrast, proprioceptive recalibration was similar between M1 and sham (*p* = 0.73). Total recalibration (sum of visual plus proprioceptive recalibration, reflecting the total magnitude participants compensated for the perturbation) was larger following S1 cTBS compared with M1 or sham (*p* = 0.012; *p* = 0.006, respectively). However, there was no difference in total recalibration between M1 and sham (*p* = 0.97). Together, these results suggest that S1 cTBS had an effect on how participants compensated for the cue conflict and that this effect was driven by proprioceptive recalibration.

#### Subjective ratings and tactile sensitivity

Subjective ratings of pain, sleep quality, attention, and fatigue were similar across groups, as was baseline neurophysiology (Table 3).

**Table 3.** Expt. 2 summary of neurophysiology and subjective ratings across groups

	M1 (mean ± 95% CI)	S1 (mean ± 95% CI)	Sham (mean ± 95% CI)
RMT (% MSO)	54.6 ± 3.4%	52.2 ± 3.4%	53.2 ± 3.2%
Attention rating	7.39 ± 0.52	7.18 ± 0.50	7.62 ± 0.51
Fatigue rating	4.07 ± 0.78	3.96 ± 0.84	3.53 ± 0.77
Sleep rating	6.78 ± 0.66	7.44 ± 0.47	6.89 ± 0.79
Pain rating	1.82 ± 0.43	1.85 ± 0.49	1.54 ± 0.33

RMT, resting motor threshold; MSO, maximum stimulator output. All ratings were on a scale from 1 to 10, reported verbally, with 10 being the best attention and sleep and the worst fatigue and pain.

An adapted GOT was used as a rough estimate of tactile sensitivity in the target index finger before and after cTBS. After cTBS, the M1 and S1 groups showed a change in GOT threshold of −0.04 ± 0.26 and 0.12 ± 0.27, respectively (mean ± 95% CI). The sham group change in GOT threshold was 0.004 ± 0.24. Change in GOT after cTBS did not differ significantly across groups (*χ*^2^ = 0.33; *p* = 0.85). The Bayes factor was calculated as 0.148, denoting substantial evidence for equivalence among the groups.

### Control experiment

**Figure 6.**
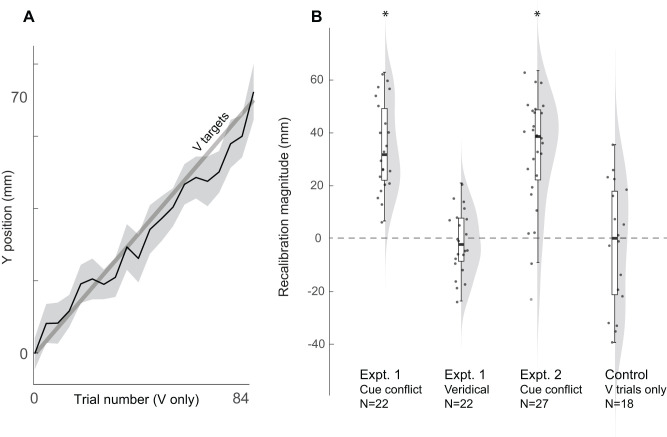
Control experiment results. ***A***, In the absence of P or VP trials, participants successfully pointed (black line) at the visual target (thick gray line) throughout the 70 mm displacement (averaged every 4 trials for clarity). Shading represents SEM. ***B***, Visual recalibration computed for the Expt. 1 cue conflict and veridical sessions (expected to be greater than and similar to zero, respectively), the Expt. 2 sham group cue conflict block (expected to be greater than zero), and the control experiment (expected to be similar to zero). Single subjects (dots), box plots (white bars), and violin plots (gray shading) are shown to illustrate each distribution. *p < 0.05.

We hypothesized that in the absence of bimodal VP trials to create a cue conflict, there would be no visual recalibration, and therefore subjects would closely follow the visual cue without undershooting it. Participants did not have any apparent difficulty pointing consistently at the V target throughout the gradual 70 mm displacement (Fig. 6*A*). The computed average change in V target undershooting was −2.32 ± 5.52 mm (mean ± SE), which was not significantly different from zero (*t*_(17)_ = −0.42; *p* = 0.68; Cohen's *d* = −0.099). Together, these results are consistent with no visual recalibration or minimization of effort, on average, despite the forward 70 mm shift of the visual target.

We also compared visual recalibration in the control experiment with how this parameter looked when there actually was a cue conflict in the two main experiments (Fig. 6*B*). As expected, visual recalibration was significantly greater than zero in the cue conflict session of Expt. 1 (*t*_(21)_ = 9.80; *p* < 0.001; Cohen's *d* = 2.09) and in the cue conflict block of Expt. 2 (*t*_(26)_ = 7.96; *p* < 0.001; Cohen's *d* = 1.53). Thus, in the presence of a 70 mm forward shift of the visual cue relative to the proprioceptive cue in bimodal trials, visual recalibration is large and easily detectable.

Finally, we examined visual recalibration in the veridical session of Expt. 1 (Fig. 6*B*). As expected, in the absence of any cue conflict or shift of the visual target, there was no significant visual recalibration (*t*_(21)_ = −0.49; *p* = 0.63; Cohen's *d* = −0.104). Descriptively, we notice that the distribution of visual recalibration is less spread out for the veridical session of Expt. 1 than for any of the comparisons, including the control experiment. This could suggest that shifting the visual target adds noise, even if the response is unbiased across subjects.

In sum, neither bimodal targets (veridical session) nor a forward-shifted visual cue (control experiment) was sufficient on its own to induce nonzero visual recalibration; this only occurred in response to a forward-shifted visual cue combined with bimodal targets, as in the cue conflict conditions.

## Discussion

We asked whether S1 conveys hand representation updates to the motor system and whether it reflects multisensory computations or only unisensory processing. Expt. 1 showed that SAI, a measure of somatosensory–motor integration, changed with proprioceptive recalibration, reflecting the results of multisensory computations. This is inconsistent with a pure hierarchical convergence model of visuoproprioceptive integration. Expt. 2 found that modulating S1, but not M1, increased proprioceptive variance and recalibration. This is consistent with the idea that motor effects of proprioceptive recalibration may be mediated by the S1→M1 pathway, although not directly controlled by M1 itself. The specificity of our findings to proprioceptive—not visual—recalibration argues against a fully distributed framework, but our findings support a model of multisensory integration with reciprocal interactions between S1 and PPC (Fig. 1*B*).

### Plasticity in S1 projections to M1 reflects proprioceptive recalibration

In Expt. 1, changes in SAI were associated with proprioceptive recalibration in the cue conflict session but not the veridical session. This effect was not present for proprioceptive estimation variance. Change in SAI being related to proprioceptive recalibration but not variance, and only in the cue conflict session, supports the idea that S1 activity represents an outcome of multisensory integration (proprioceptive recalibration). We previously found that individuals who recalibrated proprioception more had larger decreases in M1 excitability, while individuals who recalibrated vision more had larger increases in M1 excitability (Munoz-Rubke et al., 2017; Mirdamadi et al., 2022). Since the magnitude of SAI is proportional to the magnitude of somatosensory afference to S1 (Bailey et al., 2016), decreased SAI likely reflects decreased S1 excitability, which may have driven the previously observed decreases in M1 excitability in individuals who recalibrated proprioception to a greater extent (Munoz-Rubke et al., 2017; Mirdamadi et al., 2022). In contrast, the lack of association between SAI changes and visual recalibration suggests that changes in M1 excitability that we previously associated with visual recalibration are mediated by areas outside of S1, such as posterior parietal or premotor areas (Bolognini and Maravita, 2007; Hagura et al., 2007; Gentile et al., 2011; Brozzoli et al., 2012; Reichenbach et al., 2014).

Decreased influence of somatosensory afferents on M1 excitability may reflect somatosensory gating to help resolve a multisensory conflict. Decreased S1 excitability and decreased SAI has also been reported during and after the rubber hand illusion (RHI), another visuoproprioceptive cue conflict paradigm (Isayama et al., 2019). In contrast, we found no average difference in SAI after the visuoproprioceptive cue mismatch relative to veridical session. While both paradigms involve a multisensory conflict, the RHI involves synchronous stroking of a seen fake arm and felt real arm, introducing temporal visuotactile cues. In addition, the subject knows consciously that the fake arm is not their real arm (Samad et al., 2015; Chancel et al., 2022). Our participants, in contrast, were mostly unaware of the visuoproprioceptive conflict (Hsiao et al., 2022).

Besides using SAI to explore plasticity in somatosensory projections to M1 in response to a visuoproprioceptive cue conflict, baseline SAI may have functional relevance that predicts how individuals respond during the visuoproprioceptive task. We found that baseline SAI was associated with proprioceptive variance, but not visual variance, weighting, or recalibration, suggesting that baseline SAI may reflect lower-level proprioceptive processing rather than multisensory computations related to the cue mismatch. The lack of association between baseline SAI and recalibration is similar to that observed with the RHI (Isayama et al., 2019). Instead, baseline long-latency afferent inhibition was associated with the strength of the illusion, suggesting that processing in other areas like premotor and parietal cortices (Brozzoli et al., 2012; Turco et al., 2018; Guterstam et al., 2019) may be important in understanding mechanisms of multisensory integration.

### S1 activity is important for proprioceptive variance and recalibration

If previously reported modality-specific associations between recalibration and M1 excitability (Munoz-Rubke et al., 2017; Mirdamadi et al., 2022) were driven by somatosensory projections to M1, then one would predict dissociable effects of M1 and S1 neuromodulation on visuoproprioceptive recalibration. In Expt. 2, we found that S1, but not M1, cTBS affected proprioceptive variance, supporting the role of S1 in proprioceptive processing for guiding action.

S1 cTBS increased total recalibration, a difference apparently driven by proprioceptive rather than visual recalibration. However, S1 cTBS did not disrupt the normal inverse relationship between proprioceptive and visual recalibration (Ghahramani et al., 1997; Block and Bastian, 2011, 2012; Block et al., 2013). This is consistent with S1's role in proprioceptive recalibration being somewhat independent of other multisensory computations that mediate this relationship, such as parietal (Bolognini and Maravita, 2007; Hagura et al., 2007; Block et al., 2013) or prefrontal cortices (Ehrsson et al., 2004; Ehrsson and Chancel, 2019) or the cerebellum (Hagura et al., 2009). In other words, disrupting S1 did not evidently disrupt the coordination of the visual versus proprioception relationship in responding to a cue conflict. While proprioceptive variance by itself cannot adequately explain proprioceptive recalibration, which depends on both vision and proprioception, it is possible S1 cTBS affected proprioceptive recalibration indirectly, by increasing the proprioceptive variance signal coming into multisensory PPC.

The increase in proprioceptive variance and recalibration associated with S1 cTBS raises questions about potential changes in tactile function, as participants relied on feeling smooth or rough markers to place their target finger. However, S1 cTBS did not affect the GOT, indicating no confounding impact on tactile function. While prior studies reported S1 cTBS impairing tactile perception, these used small samples and a variety of methods and timing. Given the depth and focality limits of TMS (Deng et al., 2013), it likely stimulated both tactile and proprioceptive regions within S1 (Siebner et al., 2022). Importantly, the effects of S1 and M1 cTBS were dissociable on a multisensory perceptual task that guided action, the main focus of this study.

### Motor system considerations

Proprioception plays an integral role in motor control, and M1, the cortical region most associated with movement execution, is closely interconnected with S1. The neural basis of these reciprocal connections has been supported behaviorally; a visuoproprioceptive cue conflict in the absence of motor adaptation affects not only perception but also subsequent reaching movements, suggesting a common sensorimotor map for perception and action in this paradigm (Block and Liu, 2023). In other words, any changes in perceived hand position need to be considered when the brain plans hand movements. This presumably reflects a change in S1, M1, or both. It is therefore interesting to note that SAI changed in association with proprioceptive recalibration, and S1 but not M1 cTBS increased proprioceptive recalibration; this is consistent with the idea that motor effects of proprioceptive recalibration may be mediated by the S1→M1 pathway, although not directly controlled by M1 itself. This implies that our previous findings of modality-specific associations of M1 excitability with recalibration (Munoz-Rubke et al., 2017; Mirdamadi et al., 2022) were a consequence of plasticity elsewhere in the brain, and not evidence of the causal role of M1 in recalibration.

Given S1's involvement in motor planning (Asmussen et al., 2014) and skill learning (Mirdamadi and Block, 2020), we must ask if our findings could reflect motor processing rather than proprioceptive recalibration. Proprioceptive recalibration often occurs in visuomotor adaptation, where sensory prediction errors and visuoproprioceptive cue conflict drive adjustments (Rossi et al., 2021). However, visuomotor adaptation is unlikely here, as participants received no online or endpoint movement feedback. While the offset visual cue after placing the target finger on a tactile marker might resemble a sensory prediction error, this is unlikely since (1) the tactile marker itself was the explicit movement goal and (2) our prior studies indicate subjects have low certainty about the visuoproprioceptive offset in this paradigm (Hsiao et al., 2022; Hsiao and Block, 2024).

Our results align with motor learning research that suggests S1 and M1 play distinct roles in motor adaptation and retention (Kumar et al., 2019; Darainy et al., 2023). Suppressing S1, but not M1, has been shown to impair adaptation without affecting movement kinematics, indicating S1's specific role in learning mechanisms (Darainy et al., 2023). S1 is thought to contribute to the encoding and retention of learned movements (Kumar et al., 2019; Darainy et al., 2023; Ebrahimi and Ostry, 2024), motor learning by observation (McGregor et al., 2016; McGregor and Gribble, 2017), and changes in proprioception accompanying motor learning (Ostry and Gribble, 2016). If what is learned in such tasks is partly an updating of the hand representation, our findings could clarify S1's contribution to learning. S1 cTBS effects on proprioceptive variance and recalibration suggest that S1's role in motor adaptation may stem from changes in limb representation influencing motor learning.

### Visual recalibration does not reflect effort minimization

Previous studies have demonstrated both visual and proprioceptive recalibration in response to a cue conflict (Block and Bastian, 2011; Rand and Heuer, 2019), though some have suggested visual recalibration is minimal (Simani et al., 2007; Zbib et al., 2016). Visual recalibration likely depends on the weighting of visual cues, which varies by task. In the present cue conflict paradigm, participants rely more on proprioception than vision (Block and Sexton, 2020), consistent with evidence that recalibration is inversely related to cue weighting. However, we wanted to test the possibility that what we refer to as visual recalibration (increasing undershoot of the visual target) is not an artifact of introducing the cue conflict by a forward displacement of the visual cue. In other words, participants might have a general tendency to avoid moving their indicator hand farther away as the visual cue shifts, i.e., effort minimization rather than a change in perception.

The control experiment tested this with a block of visual cues gradually shifting forward, with no bimodal cues to induce a cue conflict. Participants shifted their estimates along with the shifting cue, with no increase in undershooting. Visual cue undershooting was specific to the cue conflict condition, which is inconsistent with the idea of effort minimization.

## Conclusions

Representation of the hand is critical for accurate control of movement, and our results suggest that S1 plays a key role in updating the motor system when this representation is changed by a cue conflict. S1 activity reflects the results of visuoproprioceptive computations in terms of proprioceptive but not visual recalibration, supporting reciprocal interactions with multisensory regions rather than hierarchical convergence or a fully distributed model of multisensory integration.
